# Huntingtin-Associated Protein 1 Interacts with Breakpoint Cluster Region Protein to Regulate Neuronal Differentiation

**DOI:** 10.1371/journal.pone.0116372

**Published:** 2015-02-11

**Authors:** Pai-Tsang Huang, Chien-Ho Chen, I-Uen Hsu, Shaima’a Ahmad Salim, Shu-Huei Kao, Chao-Wen Cheng, Chang-Hao Lai, Cheng-Fan Lee, Yung-Feng Lin

**Affiliations:** 1 Department of Occupational Medicine, Wan Fang Hospital, Taipei Medical University, Taipei, Taiwan; 2 School of Medical Laboratory Science and Biotechnology, College of Medical Science and Technology, Taipei Medical University, Taipei, Taiwan; 3 Graduate Institute of Clinical Medicine, College of Medicine, Taipei Medical University, Taipei, Taiwan; 4 Graduate Institute of Medical Sciences, College of Medicine, Taipei Medical University, Taipei, Taiwan; McGill University Department of Neurology and Neurosurgery, CANADA

## Abstract

Alterations in microtubule-dependent trafficking and certain signaling pathways in neuronal cells represent critical pathogenesis in neurodegenerative diseases. Huntingtin (Htt)-associated protein-1 (Hap1) is a brain-enriched protein and plays a key role in the trafficking of neuronal surviving and differentiating cargos. Lack of Hap1 reduces signaling through tropomyosin-related kinases including extracellular signal regulated kinase (ERK), resulting in inhibition of neurite outgrowth, hypothalamic dysfunction and postnatal lethality in mice. To examine how Hap1 is involved in microtubule-dependent trafficking and neuronal differentiation, we performed a proteomic analysis using taxol-precipitated microtubules from *Hap1*-null and wild-type mouse brains. Breakpoint cluster region protein (Bcr), a Rho GTPase regulator, was identified as a Hap1-interacting partner. Bcr was co-immunoprecipitated with Hap1 from transfected neuro-2a cells and co-localized with Hap1A isoform more in the differentiated than in the nondifferentiated cells. The Bcr downstream effectors, namely ERK and p38, were significantly less activated in *Hap1*-null than in wild-type mouse hypothalamus. In conclusion, Hap1 interacts with Bcr on microtubules to regulate neuronal differentiation.

## Introduction

The prevalence of neurodegenerative diseases is increasing due to an increase in aging population, but the molecular mechanism remains elusive. Neurodegenerative diseases such as Alzheimer’s disease (AD) and Huntington’s disease (HD) are characterized by progressive neuropsychiatric dysfunction and loss of specific subtypes of neurons. Although there are distinct neuropathology and clinical profiles among these diseases, many similarities in pathological pathways were reported [1]. One of the common pathogenic events in neurodegenerative diseases is impaired intracellular trafficking in neuronal cells. The critical role of intracellular trafficking in neurodegenerative diseases is also supported by mounting evidence that mutations of proteins involved in microtubule-dependent trafficking often induce neuronal pathologies including neurodegeneration [2].

Huntingtin (Htt)-associated protein-1 (Hap1), the first identified Htt-interacting protein, participates in microtubule-dependent trafficking [3–5]. Hap1 associates with microtubule-dependent motor proteins kinesin KIF5, kinesin light chain-2 and dynactin p150^Glued^ (p150) [6–8], and is involved in the transport of various proteins such as brain-derived neurotrophin factor, AD-related amyloid precursor protein and kalirin-7, a guanine nucleotide exchange factor (GEF), [6, 9–12]. Like Htt, which is critical for embryonic development, Hap1 is also essential for animal survival, as deletion of the *Hap1* gene in mouse leads to feeding defect, retarded growth, and early postnatal death [13–15]. Suppressing Hap1 expression reduces signaling through tropomyosin-related kinases, extracellular signal regulated kinase (ERK) and protein kinase B (or Akt), resulting in an inhibition of neurite outgrowth [16, 17]. However, unlike Htt that is ubiquitously expressed, Hap1 is enriched in the brain regions such as hypothalamus. Loss of Hap1 in mice leads to hypothalamic neuronal degeneration, and reductions of food-intake and body weight, which can be found in HD patients at late stages [13, 15, 18]. These findings suggest a fundamental role of Hap1 in hypothalamic neuron survival.

In murine brains, Hap1 consists of two isoforms (Hap1A and Hap1B) that differ in their C-terminal sequences [3]. The major Hap1 isoform in primate brain is more similar to murine Hap1A than to Hap1B [19]. Previous report showed the different subcellular localizations of the two isoforms [20]. Hap1A is enriched in the growth cones and neuritic puncta of developing neurons, while Hap1B is diffusely distributed within the cytoplasm. Cells overexpressing Hap1A develop more extended neurites than do those overexpressing Hap1B, indicating a dominant role for Hap1A in neuronal differentiation.

Breakpoint cluster region (Bcr) protein is enriched in neurons and involved in neural activities [21–23]. It contains tandem DH-PH which has GEF activity, and GTPase activating protein (GAP) domains to regulate Rho GTPases [24, 25]. Originally Bcr was identified as a Bcr-Abl fusion protein via Philadelphia chromosomal translocation and induces chronic myeloid leukemia [26]. Recently researchers found that Bcr-Abl fusion protein interacts with Abelson helper integration site 1 (Ahi1) [27], a protein that forms a stable complex with Hap1 in neurons [16], implicating a close relationship of Hap1 and Bcr in neuronal activities. Early studies suggested the involvement of Bcr in neural development and regulation of long-term potentiation and memory formation [21]. In Bcr-null mice, the number of dendrites was increased, indicating a dysregulation of its GAP and GEF activities in their brains [23]. In support of this, the Bcr downstream effectors are found to be the mitogen-activated protein (MAP) kinases including p38, extracellular signal regulated kinase (ERK) and c-Jun N-terminal kinase (JNK), which are implicated in cellular processes such as regulation of cell cycle, differentiation and morphogenesis [28, 29]. In addition, the activation of p21-activated kinase (PAK) was reported to correlate with Rac1 GTPase activity and is increased in Bcr-null mice [21]. In the present study, we hypothesize that Hap1 interacts with Bcr for hypothalamic neuron development.

## Materials and Methods

### Animals

*Hap1*-null mice were generated via the conventional gene knock-out approach as described [13]. The first two exons of the *Hap1* gene were deleted and replaced with a neomycin gene. Before brain excision, neonates were anesthetized on ice. All animal procedures were approved by the Institutional Animal Care and Use Committee in Taipei Medical University (LAC-99–0143).

### Cell cultures and plasmids

Neuro-2a mouse neuroblastoma cell line (ATCC, CCL-131) was maintained as monolayer cultures in 90% minimum essential medium (Eagle) with 2 mM L-glutamine, 0.1 mM non-essential amino acids, 1.5g/L sodium bicarbonate and 1.0 mM sodium pyruvate (Life Technologies Co., U.S.A), and supplemented with 10% heat inactivated fetal bovine serum (Life Technologies Co., U.S.A), 100 units/mL penicillin, 100 μg/mL streptomycin, and 2.5 μg/mL amphotericin B (Life Technologies Co., U.S.A). Cultures were maintained at 37°C with 5% CO_2_ in a humidified incubator. For immunofluorescence experiment, the cells were cultured in slide chambers (Merck Millipore, U.S.A.). The cells were transfected with 1 μg of DNA according to the manufacturer protocol of TurboFect (Thermo Scientific, U.S.A.) as required.

Plasmids used in this study were a green fluorescent protein (GFP) fusion vector, pEGFP-C3 (BD Biosciences Clontech, U.S.A.), GFP-fused rat *Hap1* constructs, pEGFP-rHap1A and pEGFP-rHap1B [6], and myc-tagged mouse Bcr construct, pCMV6-mBcr-myc (OriGene, U.S.A.).

### Antibodies

Rabbit anti-JNK polyclonal, rabbit anti-p38 polyclonal, mouse anti-phosphorylated JNK (p-JNK) Thr183 and Tyr185 monoclonal, mouse anti-PAK monoclonal, mouse anti-p-p38 Tyr182 monoclonal, and goat anti-Hap1 (N-18) polyclonal antibodies were purchased from Santa Cruz, U.S.A. Rabbit anti—Bcr polyclonal, anti-p-PAK1/2 Ser144/141 polyclonal, anti-p-ERK1/2 Thr202/204 monoclonal, and mouse anti-ERK1/2 monoclonal antibodies were purchased from Cell Signaling, U.S.A. Mouse anti—Myc antibody was purchased from OriGene Technologies, U.S.A. Rabbit anti-GFP polyclonal and secondary antibodies conjugated with HRP, Dylight 488 and Dylight 594 were purchased from GeneTex, Taiwan.

### Microtubule sedimentation and LC-MS/MS

A wild-type C57BL/6 female mouse was fed with heavy isotope-labeled chow containing [^13^C_6_^15^N_4_] arginine and [^13^C_6_] lysine before pregnancy. Its offspring kept the same diet to adulthood. Brain lysates from the isotope-labeled wild-type (WT) adult mouse and a non-labeled newborn mouse at P1, with either WT or *Hap1*-null genotype, were mixed at 1:1 ratio in a buffer containing 50 mM PIPES pH 6.8, 1 mM EGTA, 1 mM MgSO_4_ and complete protease inhibitor cocktail (Roche). Microtubule-associated proteins were polymerized by taxol (Sigma) as described previously [30]. Briefly, 200 μl of the mixed brain lysates were centrifuged at 18,000 × g for 20 min, and the supernatant (S2) was further centrifuged at 120,000 × g for 30 min. The resulting supernatant (S3) was treated with 1 mM GTP and 20 μM taxol at room temperature for 30 min. The polymerized microtubules were pelleted by centrifugation at 120,000 × g for 30 min. The pellets were washed once with PIPES buffer and resuspended in 100 μl of RIPA buffer containing 10 mM dithiothreitol. The samples were resolved on a 10% polyacrylamide SDS gel. After staining with Coomassie G-250, each gel lane was cut into pieces and subjected to in-gel digestion and LC-MS/MS analysis as described [31]. Another set of microtubule sedimentation without isotope-labeled WT adult mouse brain was performed and analyzed by Western blotting to confirm the findings in mass spectrometry. Each sample with the same volume (10 μl) instead of mass was analyzed to reflect a similar level of microtubules in the lanes on the blot. S2 supernatant was used as input.

### Immunofluorescence imaging

Neuro-2a cells were cultured in 4-well slide chamber (Merck Millipore, U.S.A.) at 10,000 cells per well. Cells were co-transfected with pCMV6-Bcr-myc and pEGFP-C3, pEGFP-Hap1A or pEGFP—Hap1B. After transfection, cells were washed with 1X PBS and fixed using 4% paraformaldehyde for 5 min at room temperature. Following washes, cells were permeablized and blocked with 0.1% Triton X-100, 3% BSA in PBS for 30–60 min at room temperature. Afterward the cells were incubated with primary mouse anti-Myc antibody overnight. The cells were then washed and incubated with a fluorophore-conjugated secondary antibody at 1:5000 for 2 hours at room temperature. After washes, the cells were incubated with 300 ng/ml DAPI in 1X PBS for 10 min at room temperature. With sufficient washes, the chamber was disassembled, and the slide was air-dried and mounted. The slide was covered and sealed, and then visualized using TCS SP5 Confocal Spectral Microscope Imaging System (Leica, Germany) at Taipei Medical University Instrument Center. Images of co-localized area were generated using Image J 1.48v (Wayne Rasband, NIH, U.S.A), and image densities were quantified using Un-Scan-It 6.1 (Silk Scientific Crop., U.S.A.).

### Immunoprecipitation

Neuro-2a cells were harvested 48 h after transfection, and homogenized in 50 mM PIPES pH 6.8, 1 mM EGTA, 1 mM MgSO_4_, 100 μg/ml PMSF, 1X protease inhibitors and phosphatase inhibitors (Merck Millipore, U.S.A.). The homogenate was centrifuged at 15,000 rpm for 15 min at 4°C. Anti-GFP antibody was added to protein A magnetic beads (Recenttec, Taiwan) and incubated for 2 h at 4°C to immobilize the antibody. Then 300 μg of the centrifuged supernatant was incubated with immobilized antibody overnight with continuous mixing at 4°C. The immunoprecipitates were washed five times with the PIPES buffer containing 0.1% NP-40 and then eluted by boiling in SDS—PAGE sample loading buffer. The proteins were resolved on a 10% SDS-PAGE and transferred onto PVDF membrane (BioRad) for immunoblotting.

### Hypothalamus excision

Three different sets of wild type and *Hap1-null* newborn mouse (P1) hypothalamus were excised on ice. The excised tissues were homogenized and sonicated in lysis buffer (50 mM Tris, pH 7.5, 150 mM NaCl, 1% NP-40, 0.5% Sodium deoxycholate, 0.1% SDS, 1 mM DTT, 1X protease inhibitors and phosphatase inhibitors; Merck Millipore, U.S.A.) at 4°C and kept on ice through all the process.

### Western blotting

Protein concentrations of cell lysates were determined by DC protein assay kit protocol (BioRad, U.S.A.). About 20 μg of proteins were analyzed in 10% SDS-PAGE at a constant voltage of 80 V until full separation. The proteins were transferred to PVDF membrane under 300 Am for 2 h at 4°C. After transfer, the membrane was blocked using 5% nonfat milk in 1X PBS/0.05% tween-20 (PBST) for 1 h at room temperature. The membrane was washed and incubated with specific primary antibody diluted in 3% BSA PBST for overnight at 4°C. After washes, the membrane was incubated with HRP-conjugated secondary antibody diluted in 5% nonfat milk PBST for 1 h at room temperature. The membrane was washed and developed using ECL chemiluminescence kit (Thermo Scientific, U.S.A.). Quantitative analysis was done using Un-Scan-It 6.1 (Silk Scientific Crop., U.S.A.).

### Statistical analysis

All experiments were performed at least three times. Results were expressed as mean ± standard error, and analyzed through Student’s t test or one-way ANOVA using SigmaPlot 12.5 software. Values of *p* < 0.05 (*), *p* < 0.01 (**) and *p* < 0.001 (***) were considered statistically significant.

## Results

### Decreased association of Bcr with microtubules in *Hap1*-null mouse brain

**Fig 1:**
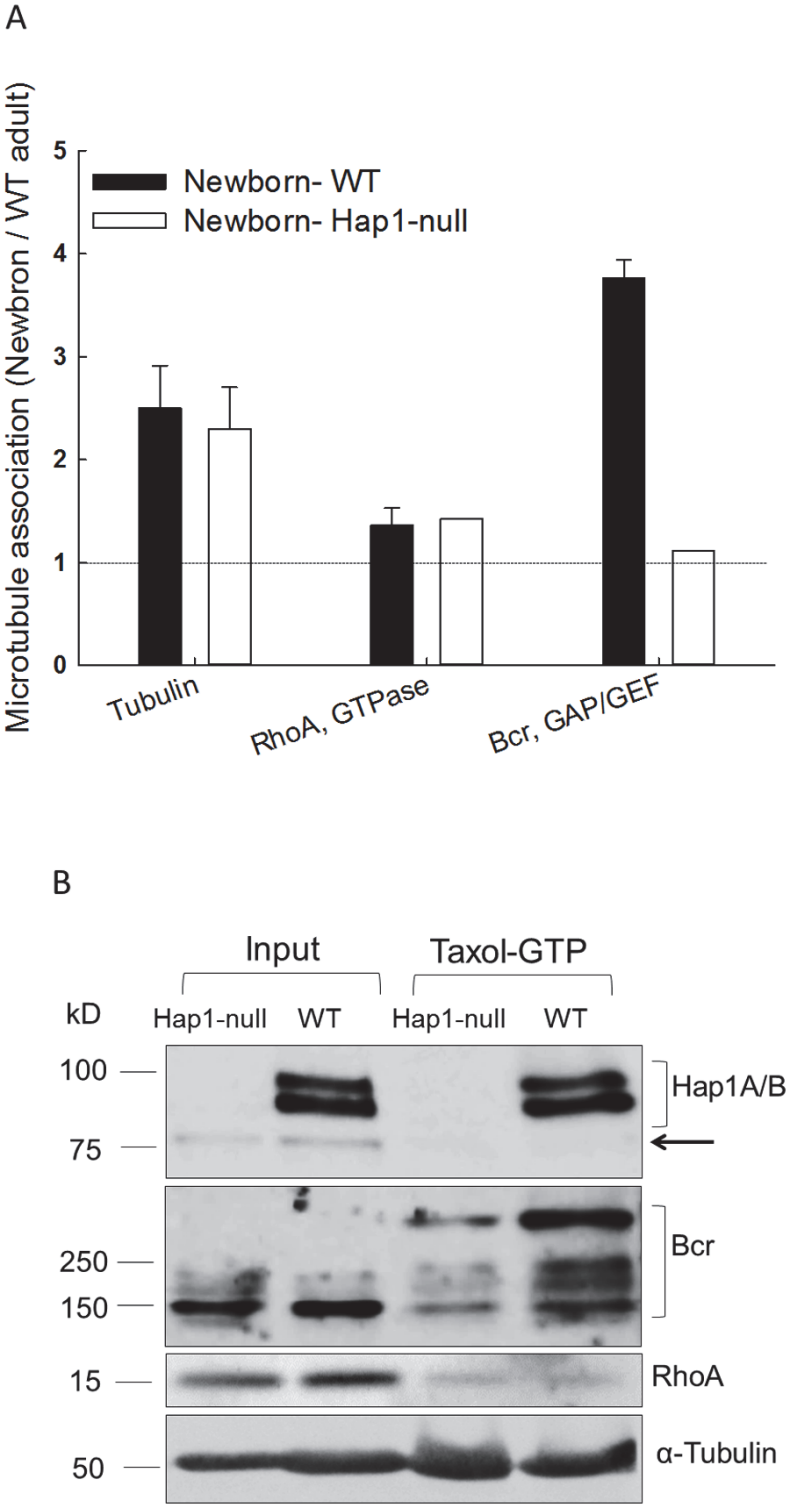
Association of Bcr with Hap1 on microtubules in mouse brains. (**A**) The relative amount of a microtubule subunit (β-tubulin), a small GTPase (RhoA) and a GAP/GEF (Bcr) in wild-type (WT) and *Hap1*-null newborn mouse brains normalized to isotope-labeled counterparts in wild type adult mouse brain. (**B**) Western blotting showing Hap1 isoforms, Bcr, RhoA and α-Tubulin in microtubule pellets precipitated by taxol-GTP treatment from *Hap1*-null and WT mouse brains. Input is the supernatant of brain lysates after centrifugation at 18,000 × g for 20 min (S2). The nonspecific protein species reacting to Hap1 antibody in the inputs (arrow) was not precipitated with microtubules.

Many known Hap1-interacting proteins are associated with microtubules. Hap1 may assist microtubule-dependent transport of specific cargos to promote neuronal differentiation. The mass spectrometry results showed higher levels of the tubulin and microtubule-associated Bcr in WT newborn brain than in adult brain, indicating the requirement of these proteins in neural development (Fig. 1A). There was a decreased association of Bcr with microtubules in *Hap1*-null mouse brain. This decrease may be a cause of the neuronal dysfunction in *Hap1*-null mice. The levels of the tubulin and a known Bcr-regulating small GTPase, namely RhoA, remained unchanged in the brain of *Hap1*-null newborn mouse. These results support a specific relationship of Bcr and Hap1 in neuronal development.

Although the mass spectrometry data should be reliable, we verify them by Western blotting with antibodies against Hap1, Bcr, RhoA and α-tubulin. Bcr levels in the input were similar in both WT and *Hap1*-null mouse brains (Fig. 1B). Indeed, Bcr was precipitated with microtubules by taxol from WT brain lysates, and this precipitation was largely reduced in *Hap1*-null brain, suggesting the requirement of Hap1 for Bcr to be associated with microtubules. In contrast, only a small portion of RhoA was associated with microtubules, and the association was not Hap1-dependent. A nonspecific protein species (~75 kD) reacting to Hap1 antibody in the input (S2) was not precipitated with microtubules, indicating an exclusion of certain proteins in the microtubule proteome. Although more tubulins were expected to be seen in taxol-precipitated samples, perhaps a portion of tubulins were lost during the sample processing.

### Co-precipitation of Bcr with Hap1 from neuronal cells

**Fig 2:**
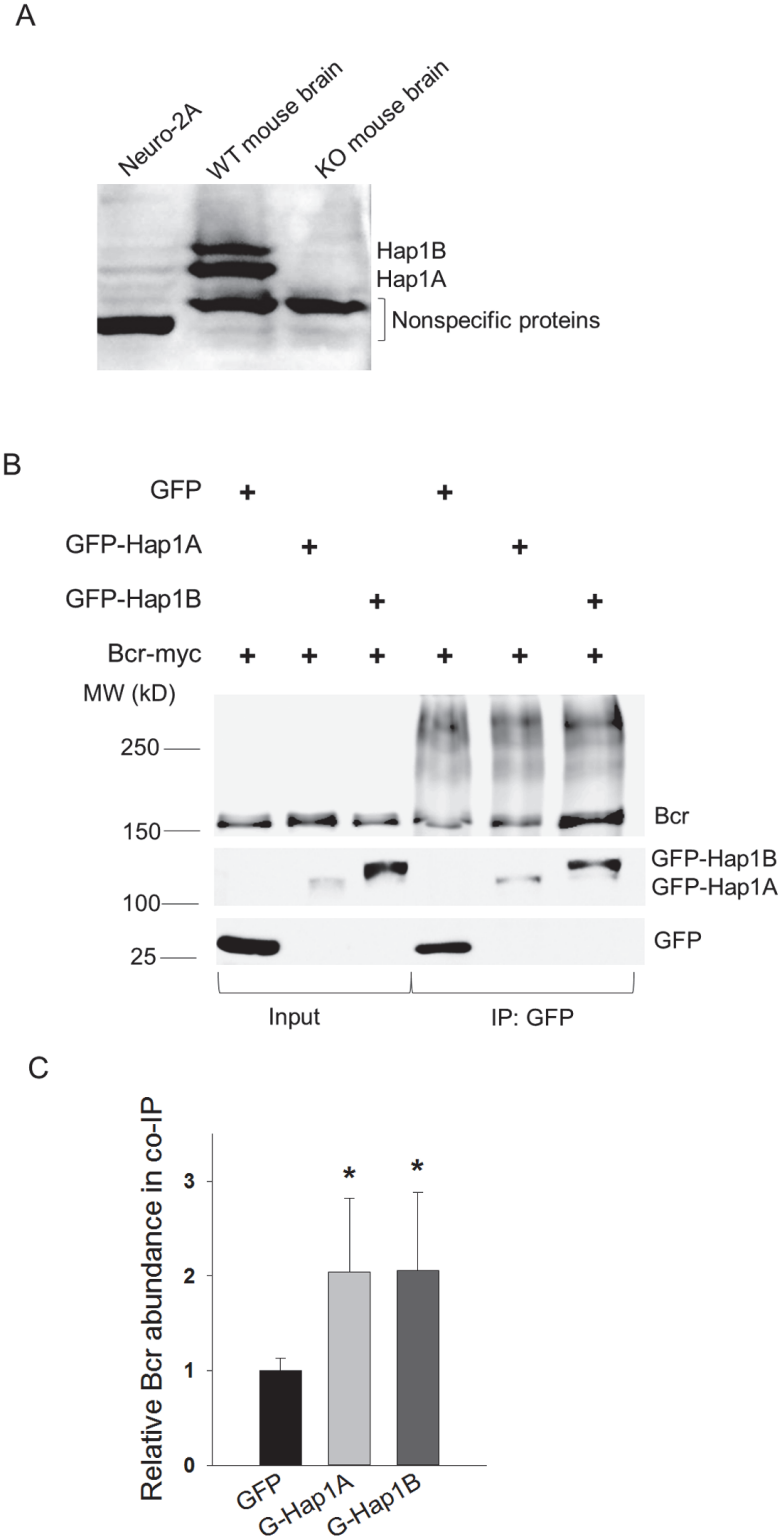
Co-immunoprecipitation of Hap1 and Bcr from neuro-2A cells. (**A**) The protein levels of Hap1A and Hap1B in the mouse neuroblastoma neuro-2a cells and total brain lysates from wild-type (WT) and *Hap1*-null mice. The nonspecific protein in each lane implicated an equal loading of the samples. (**B**) Immunoprecipitation (IP) of neuro-2a cells transfected with GFP, GFP-Hap1A, or GFP-Hap1B with Bcr-Myc. The cell lysates were precipitated with anti-GFP antibody. The input and precipitates were analyzed using SDS-PAGE and immunoblotted (IB) with anti-Bcr and anti-GFP antibodies. (**C**) Quantitative analysis demonstrating the binding of Bcr to Hap1. The Bcr abundance was normalized with the bait (GFP, Hap1A-GFP or Hap1B-GFP), and the result in GFP was set as 1. Four independent experiments were performed. * p < 0.05.

We were not able to successfully precipitate Bcr and Hap1 from mouse brain tissues using the commercially available anti-Bcr or anti-Hap1 antibodies. Owing to the inability of the antibody we used for immunoprecipitation, we performed the assay using lysates from neuro-2a cells co-transfected with Bcr-myc and GFP-Hap1. The endogenous Hap1 isoforms were expressed at low levels in neuro-2a cells compared to those in wild-type mouse brain (Fig. 2A), making neuro-2a an ideal experimental model for seeing exogenous Hap1’s overexpression. Note that the nonspecific protein species shown on Hap1 immunoblot was largely reduced in the supernatant of mouse brain lysates after high-speed centrifugation and completely disappeared in the microtubule sediments (Fig. 1B). Anti-GFP antibody was used to precipitate the GFP or GFP-Hap1 in neuro-2a cells (Fig. 2B). Precipitation of GFP-Hap1A and GFP-Hap1B were both able to pull down Bcr with significant difference from GFP precipitation, indicating a close relationship between Bcr and Hap1 (Fig. 2C).

### Co-localization of Bcr with Hap1 in differentiated neuronal cells

**Fig 3:**
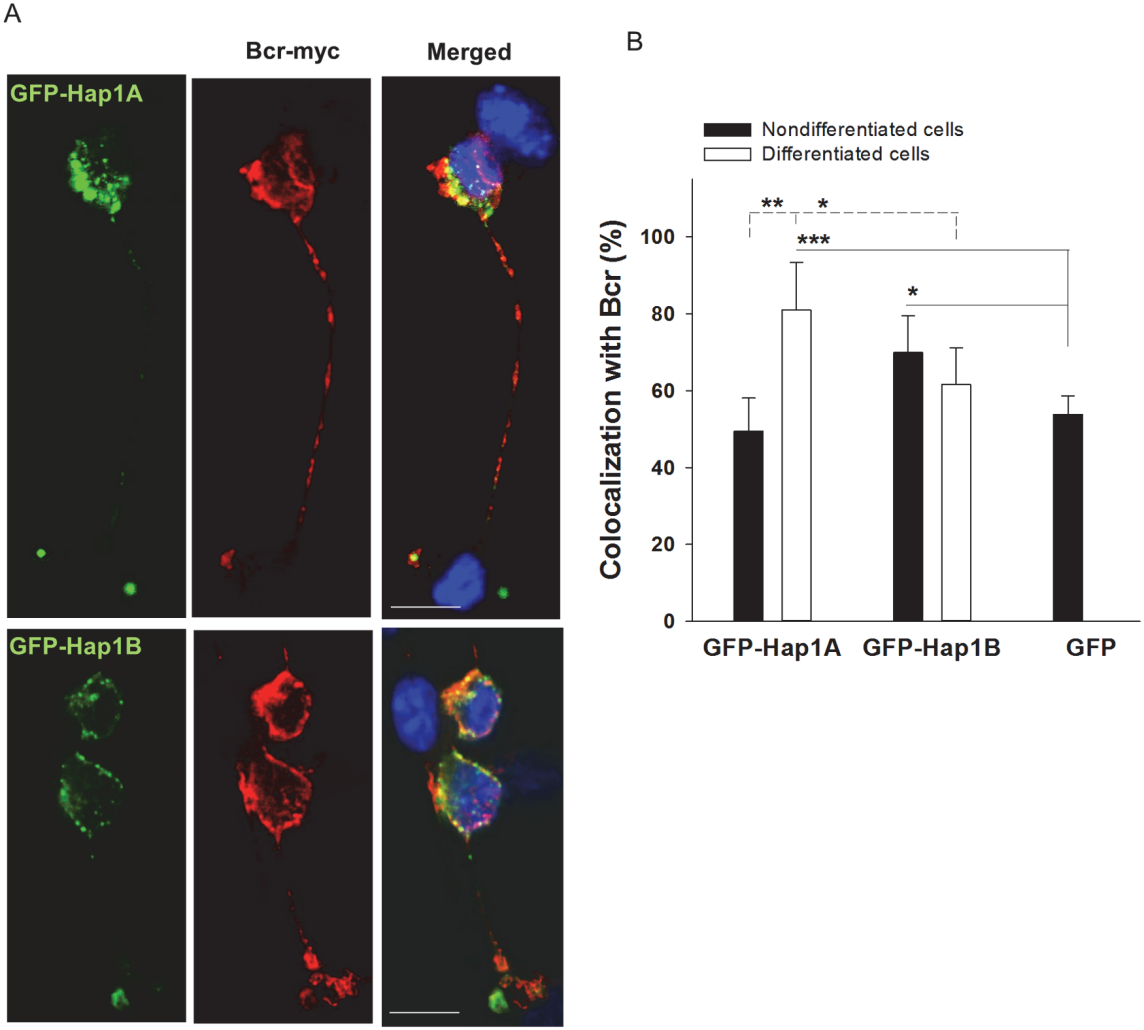
Co-localization of Bcr and Hap1 in differentiated neuro-2A cells. (**A**) Confocal imaging of differentiated neuro-2a cells transfected with GFP-Hap1A or GFP-Hap1B (green) with Bcr-myc (red). Scale bar, 10 μm. (**B**) Statistical analysis showing the percentage of GFP, GFP-Hap1A and GFP-Hap1B co-localized with Bcr in differentiated cells. Five or more sets of image were analyzed. * p < 0.05; ** p < 0.01; *** p < 0.001.

The cells expressing GFP-Hap1A or GFP-Hap1B were differentiated with obvious process extensions (S1A Fig.). The transfected cells with processes longer than their two-cell body size were quantified. Less than 2% of GFP-expressing cells differentiated with long processes, while the percentage of differentiated cells expressing GFP-Hap1A (15±1%) or GFP-Hap1B (10±1%) was significantly increased (S1B Fig.). These results are consistent with early findings using a different cell line and support a role for Hap1, especially Hap1A, in neuronal differentiation. To confirm the association of Bcr with Hap1, we co-transfected Neuro-2a cells with GFP-Hap1 and Bcr-myc. In the confocal images, GFP alone was diffused allover in the cytoplasm and nucleus, and was not obviously co-localized with Bcr (S2 Fig.). GFP-Hap1A formed inclusion-like structures (or puncta) with little co-localization with Bcr, while GFP-Hap1B formed smaller puncta with a portion of Bcr co-localized in nondifferentiated cells. In contrast, most GFP-Hap1A puncta were co-localized with Bcr in the cytoplasm and processes of differentiated cells, while GFP-Hap1B was mostly localized in the cytoplasm with a portion of co-localized Bcr (Fig. 3A). Statistical analysis indicated a stronger interaction of Bcr with Hap1A (81±12%) than with Hap1B (61±10%) in differentiated cells (Fig. 3B). These results indicate the association of Bcr with Hap1 in the same protein structure and support distinguished functions of Hap1A and Hap1B in neuronal differentiation.

### Inhibition of Bcr related signaling in *Hap1*-null mouse hypothalamus

**Fig 4:**
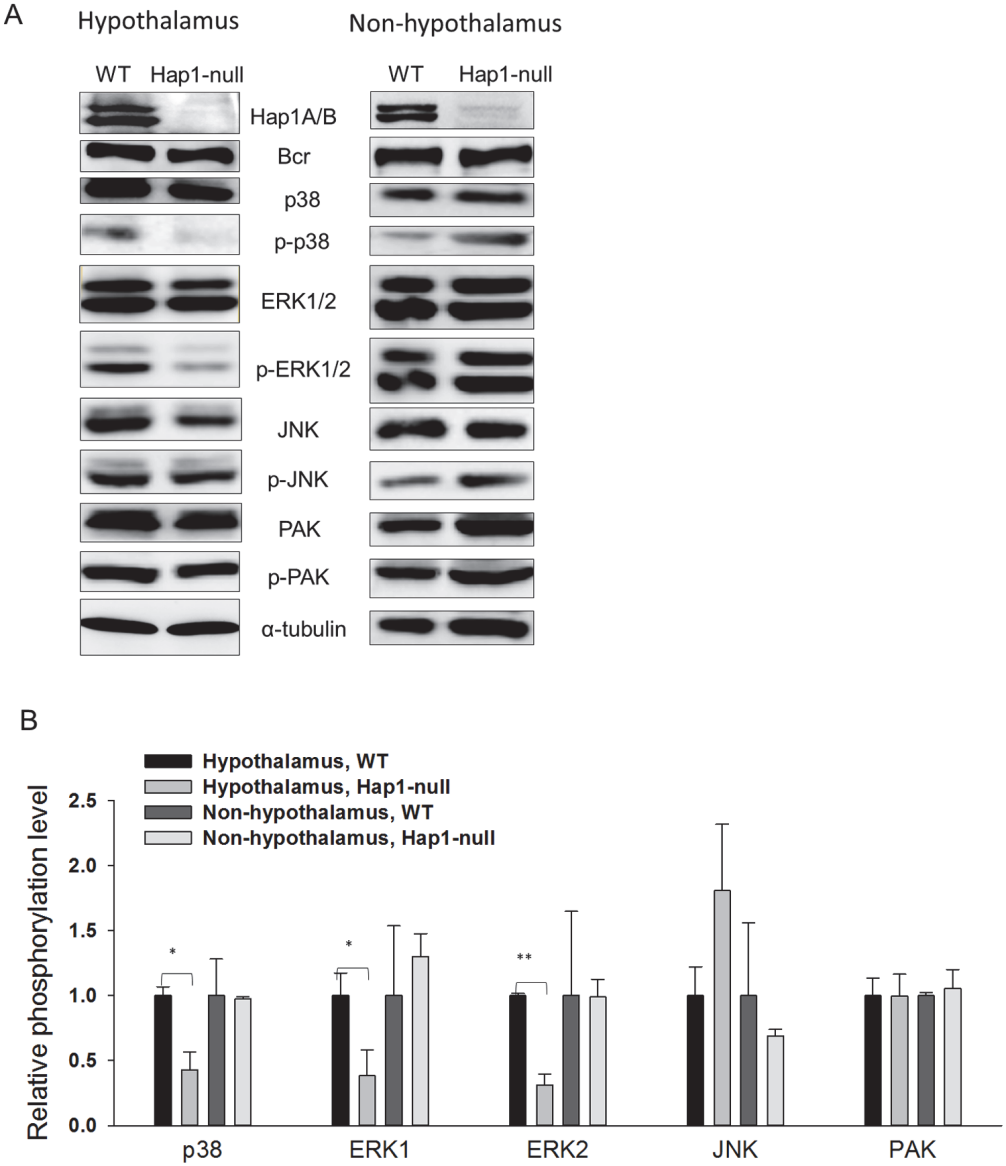
Lack of Hap1 inhibits Bcr signaling in mouse hypothalamus. (**A**) Western blotting analysis of Bcr and downstream signaling molecules including p38, ERK1/2, PAK, JNK and their phosphorylated forms from the WT and *Hap1*-null mouse hypothalamic (left panel) and non-hypothalamic regions (right panel). (**B**) Quantitative and statistical analysis of the changes of Bcr and its downstream signaling molecules in the hypothalamic and non-hypothalamic regions. The presented value was the ratio of the phosphorylated protein level to the total protein level and normalized with the result in WT mouse hypothalamic or non-hypothalamic region, which was set as 1. Three independent experiments were performed for statistical analysis. * p < 0.05; ** p < 0.01.

We wanted to examine if the lack of Hap1 can affect Bcr-related signaling in the hypothalamus, as Hap1 is enriched in hypothalamic neurons [13]. We analyzed the levels of Bcr, and those downstream effectors including the three MAP kinases (e.g. p38, ERKs and JNK) and PAK using newborn mouse hypothalamus (Fig. 4A, left panel). When compared to WT brains, the total levels of Bcr and its downstream targets were unchanged in *Hap1*-null hypothalamus; however, their activated forms including p-p38 and p-ERKs were significantly reduced in *Hap1*-null hypothalamus (Fig. 4B). Moreover, these changes were not seen in other mouse brain regions (a mixture of cortex, striatum, cerebellum and brain stem) excluding the hypothalamus (Fig. 4A, right panel and 4B). These results suggest that the interaction between Hap1 and Bcr is involved in neuronal signaling that is important for hypothalamic development.

To examine whether Hap1 affects Bcr-related signaling in cultured cells, we analyzed the levels of Bcr, and several downstream effectors involved in cell differentiation using GFP-Hap1-expressed cell lysates (S3A Fig.). When compared to control cells expressing GFP, the total levels of Bcr and its downstream targets were not changed in GFP-Hap1A or GFP-Hap1B expressed cells (S3B Fig.); however, the activated form of ERK1 (p-ERK1) was significantly increased both in the cells expressing GFP-Hap1A and GFP-Hap1B. These results support the importance of Bcr-Hap1 interactions in neuronal differentiation.

## Discussion

In this study, we provide evidence that Hap1 associates with Bcr. The interaction of Hap1 with Bcr was initially identified via mass spectrometry analysis of associated proteins with microtubules precipitated by taxol and verified by immunoprecipitation of these two proteins (Fig. 1). This finding further supports the idea that Hap1 is involved in microtubule-dependent transport. In addition, our findings suggest that Hap1 is important for Bcr-related signaling activation in the hypothalamus, as the lack of Hap1 selectively reduces the association of Bcr on microtubules (Fig. 1) and its signaling in the hypothalamic region in mice (Fig. 4).

Hap1 is likely to interact with different partners to participate in a variety of important cellular functions in different brain regions. Previous studies show that Hap1 interacts with Ahi1 to regulate cerebellar and brainstem development [16]. Other studies on mice lacking Hap1 show disruption of neuronal processes and death in hypothalamic neurons, resembling neurodegeneration in HD [13–15, 18]. A recent study shows that Hap1 is important for postnatal neurogenesis by stabilizing TrkB in mouse hypothalamus [32]. Now our data suggest a novel pathway that Hap1 interacts with Bcr in hypothalamic neuron differentiation. Although studies implicate possible involvement of Ahi1 in the interaction of Hap1 and Bcr, the microtubule deposits from neither WT nor Hap1-null mouse brain display Ahi1 in the MS result (data not shown).

Bcr was shown to activate Ras and RhoA activities with GEF function in cell proliferation and differentiation [25, 33]. In addition, Bcr also regulates synaptic Rac1 GTPase signaling and neuronal development with GAP activity [21, 23]. The downstream effectors of Bcr including p38, ERK and JNK MAP kinases are implicated in the regulation of cell cycle, gene expression, cytoskeletal actin assembly, neurite outgrowth and differentiation [28]. Bcr may interact with different proteins to regulate cell proliferation and differentiation. In our study, mice deficient for Hap1 displayed markedly reduced activation of ERK and p38 with enhanced activation of JNK, though not significantly, suggesting that Hap1 mediates the function of Bcr for neuronal survival and differentiation. Since Hap1 is abundant in the hypothalamus and defective Bcr-related signaling was not found in the brain regions excluding the hypothalamus, our studies suggest that the interaction of Hap1 with Bcr and the related signaling may be particularly important for the hypothalamic function. Such specific function would help us to further investigate how hypothalamic neurons play important roles in a variety of critical body functions such as feeding intake and growth.

Previously researchers identified the interaction of Hap1 with Trio-like GEF (Kalirin-7), a Rac1 GTPase activator regulating cell growth and dendritic spine remodeling [10, 34]. Our present proteomic analysis revealed that Bcr, another GEF, may require Hap1 for their association with microtubules in brain cells (Fig. 1). Each GEF activates a group of GTPases by stimulating the release of GDP and binding of GTP. Bcr was shown to activate Ras and RhoA activities with GEF function in cell proliferation and differentiation [25, 33]. In addition, Bcr also regulates synaptic Rac1 GTPase signaling and neuronal development with GAP activity [21]. Mice deficient for Bcr show enhanced basal activity of Rac1 and activation of PAK and ERK without affecting p38 and JNK in spine remodeling. A dominant GAP activity of Bcr was suggested in the synaptic activities [21]. In contrast in our study, mice deficient for Hap1 in the hypothalamus displayed enhanced activation of JNK, although not significantly, but reduced activation of p38 and ERK dramatically, suggesting that Hap1 mediates Bcr differentially, perhaps, via the GEF activity of Bcr for neuronal survival and differentiation (Fig. 4) [35]. It was shown that transglutaminase can cross-link Bcr and regulate Bcr GAP activity [36, 37]. Interestingly in our study, Bcr was polymerized like cross-linked species during microtubule sedimentation and immunoprecipitation (Figs. 1B and 2B). The involvement of Hap1 in the mediation of GEF and GAP activities in neurons may be worth of further investigation.

In neuronal and transfected cells, the Hap1A isoform tends to form inclusion-like puncta which were distributed in the cell body and processes (Fig. 3A) [38]. These Hap1A puncta are enriched in neurite tips and participate in neuronal differentiation [20]. Here we showed that Bcr bound to Hap1A in the puncta in where Bcr may be activated for neuronal development signaling (Fig. 4). Indeed, as demonstrated in this study, Hap1 interacts with Bcr to regulate neuronal differentiation.

## Supporting Information

S1 Fig Hap1 expression promotes neuro-2A cell differentiation. (**A**) Micrographs (phase images in the upper panel and fluorescent images in the lower panel) showing neuro-2a cells expressing GFP, GFP-Hap1A or GFP-Hap1B after 48 h of transfection. Note that cells expressing GFP-Hap1A or GFP-Hap-1B display long neurites. (**B**) Statistical analysis indicating the percentage of differentiated cells with process extension more than two-fold their cell bodies. The number of cells examined was at least 100 in each group in three independent experiments. *** *p* < 0.001.
(TIF)

S2 Fig Confocal imaging of nondifferentiated neuro-2a cells. The cells were transfected with GFP, GFP-Hap1A or GFP-Hap1B (green) with Bcr-myc (red). Scale bar, 10 μm.
(TIF)

S3 Fig Effects of Hap1 expression on Bcr downstream effectors in neuro-2A cells. (**A**) Western blotting analysis of neuro-2a cells transfected with GFP, GFP-Hap1A, or GFP-Hap1B. After 48 hours of transfection, cells were harvested and homogenized for the analysis. Antibodies against Bcr and downstream signaling molecules including p38, ERK1/2, JNK, PAK, and their phosphorylated forms were used. (**B**) Quantitative and statistical analysis of the changes ERK1 in the transfected cells. Three independent experiments were performed for statistical analysis. * *p* < 0.05
(TIF)
