# Supplementary figures and images for: Huntingtin-Associated Protein 1 Interacts with Breakpoint Cluster Region Protein to Regulate Neuronal Differentiation
Source: PLoS One. 2015 Feb 11;10(2):e0116372. doi: 10.1371/journal.pone.0116372 (PMC4324908; doi:10.1371/journal.pone.0116372)

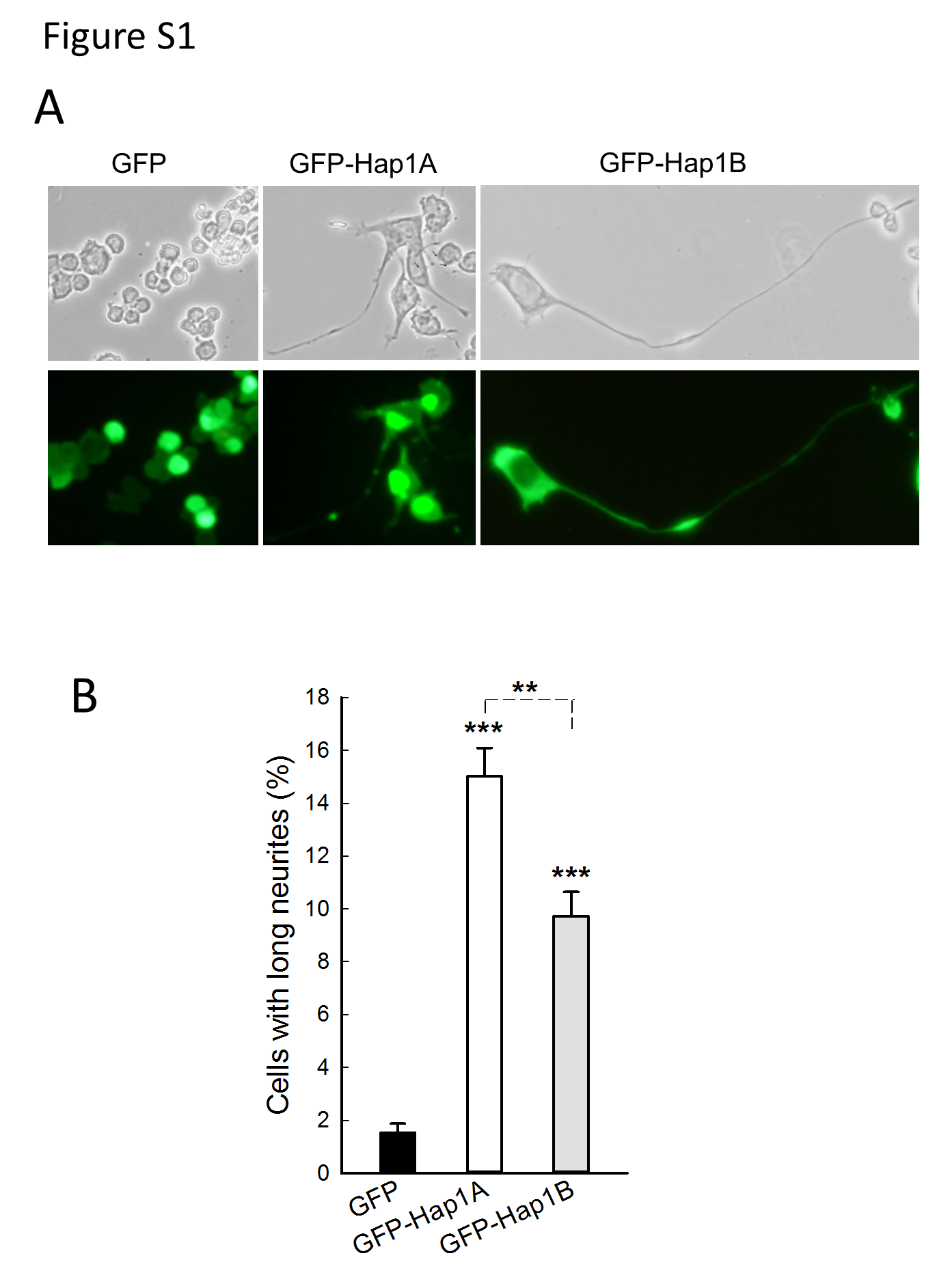

Supplement: S1 Fig — (A) Micrographs (phase images in the upper panel and fluorescent images in the lower panel) showing neuro-2a cells expressing GFP, GFP-Hap1A or GFP-Hap1B after 48 h of transfection. Note that cells expressing GFP-Hap1A or GFP-Hap-1B display long neurites. (B) Statistical analysis indicating the percentage of differentiated cells with process extension more than two-fold their cell bodies. The number of cells examined was at least 100 in each group in three independent experiments. *** p < 0.001. (TIF) [file pone.0116372.s001.tif]

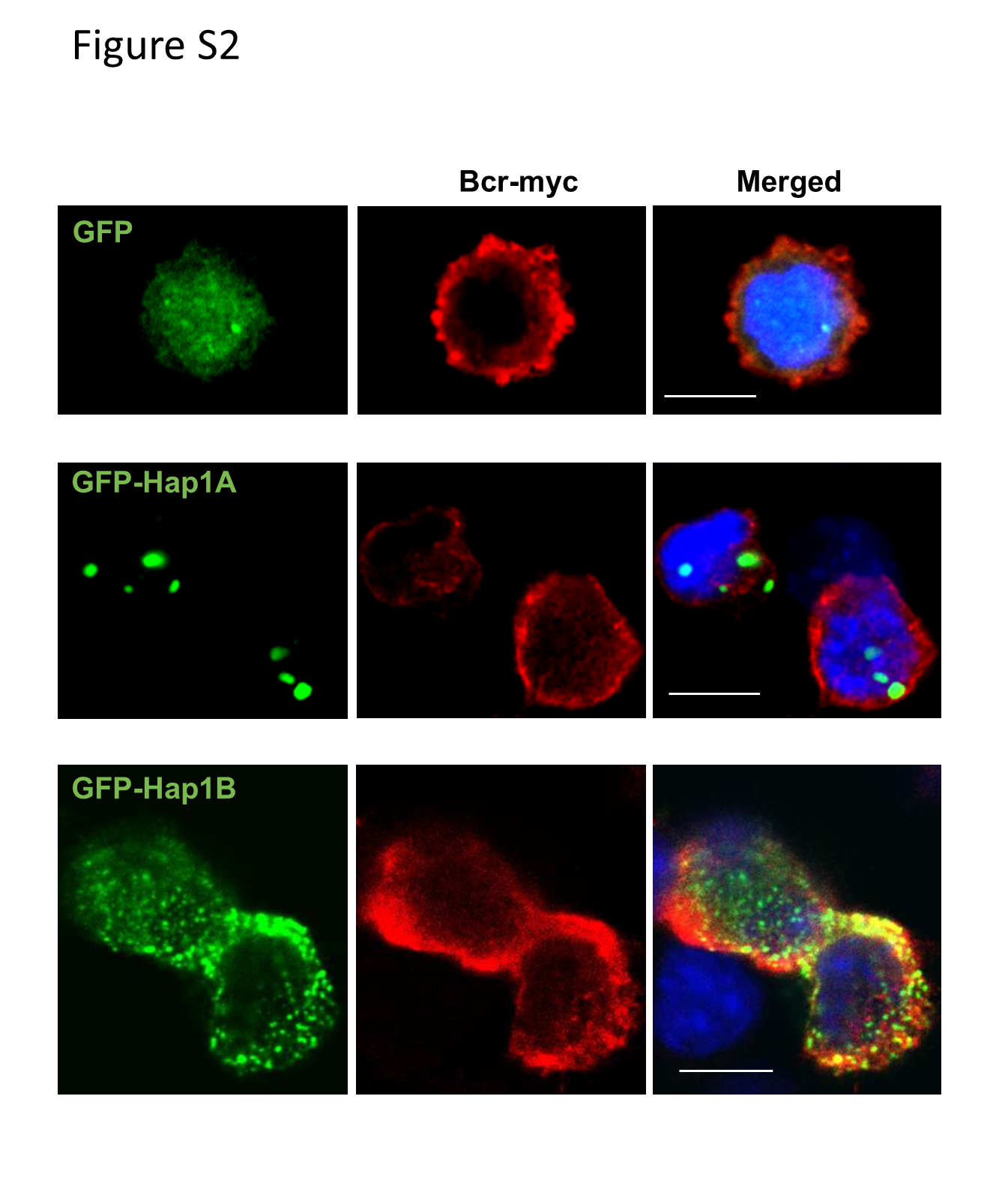

Supplement: S2 Fig — The cells were transfected with GFP, GFP-Hap1A or GFP-Hap1B (green) with Bcr-myc (red). Scale bar, 10 μm. (TIF) [file pone.0116372.s002.tif]

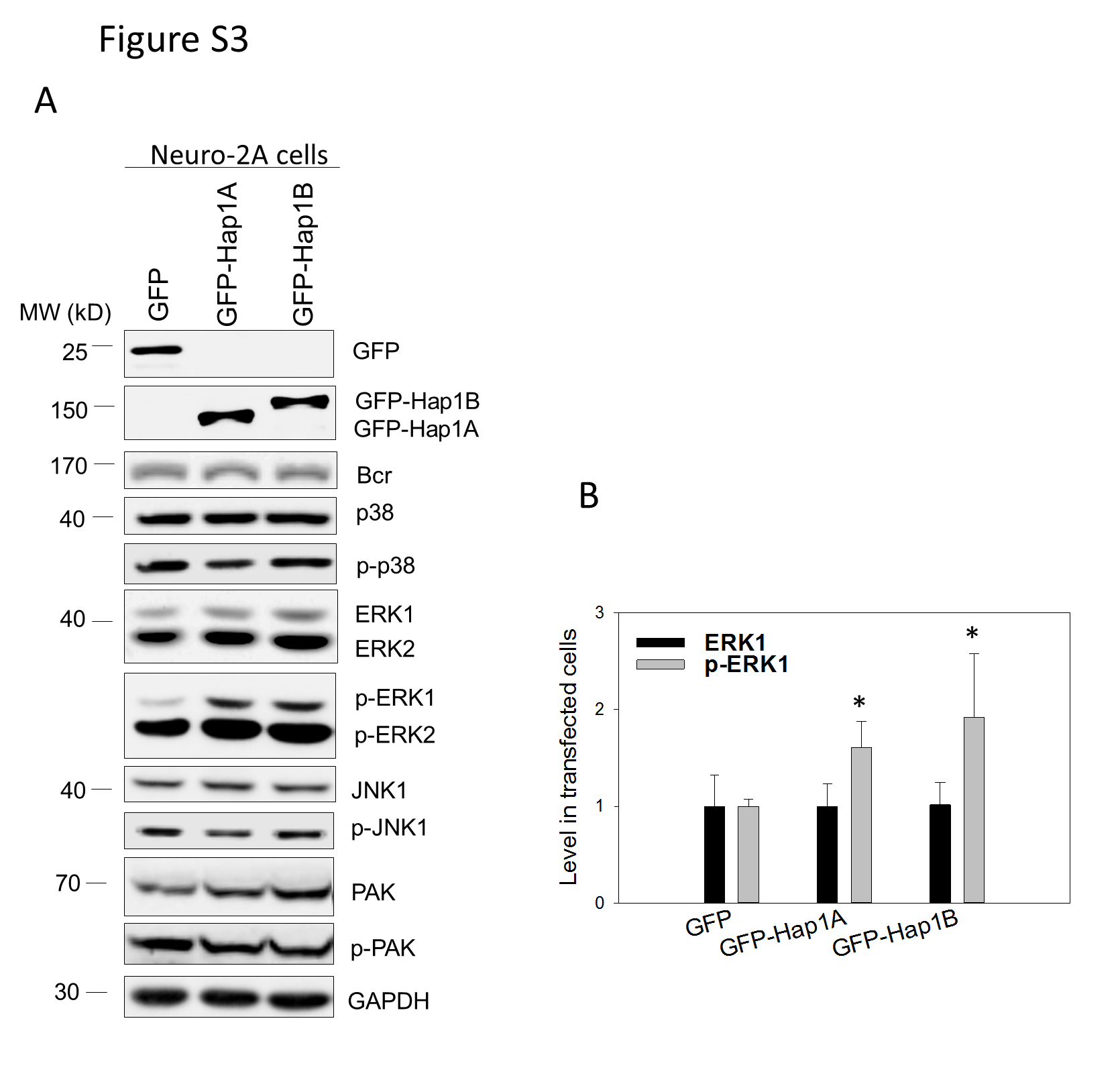

Supplement: S3 Fig — (A) Western blotting analysis of neuro-2a cells transfected with GFP, GFP-Hap1A, or GFP-Hap1B. After 48 hours of transfection, cells were harvested and homogenized for the analysis. Antibodies against Bcr and downstream signaling molecules including p38, ERK1/2, JNK, PAK, and their phosphorylated forms were used. (B) Quantitative and statistical analysis of the changes ERK1 in the transfected cells. Three independent experiments were performed for statistical analysis. * p < 0.05 (TIF) [file pone.0116372.s003.tif]
